# Complete Genome Sequences of *Microbacterium* Bacteriophages Danno, Otwor, and Scumberland, Isolated in Clarksville, Tennessee

**DOI:** 10.1128/MRA.00209-21

**Published:** 2021-04-01

**Authors:** Sergei A. Markov, James C. Church, Leong Lee, Cole M. Bell, Sarah D. Binkley, Kevin M. Bouma, Kaitlin M. Hutson, Gregory S. Markov, Elizabeth C. Mason, Gabrielle B. Rueff, Taiwo O. Sennuga, Montana H. Simpson, Robin J. Zimmer, Diana G. Villalpando

**Affiliations:** a Biology Department, Austin Peay State University, Clarksville, Tennessee, USA; b Computer Science and Information Technology Department, Austin Peay State University, Clarksville, Tennessee, USA; Queens College CUNY

## Abstract

This paper reports the genome sequences of bacteriophages isolated from soil samples using Microbacterium foliorum. Phages Danno and Otwor (cluster EE) have genomes of 17,452 bp and 17,454 bp, respectively, and 25 predicted genes. The phage Scumberland (cluster EC) has a genome of 53,276 bp with 92 predicted genes.

## ANNOUNCEMENT

**FIG 1:**
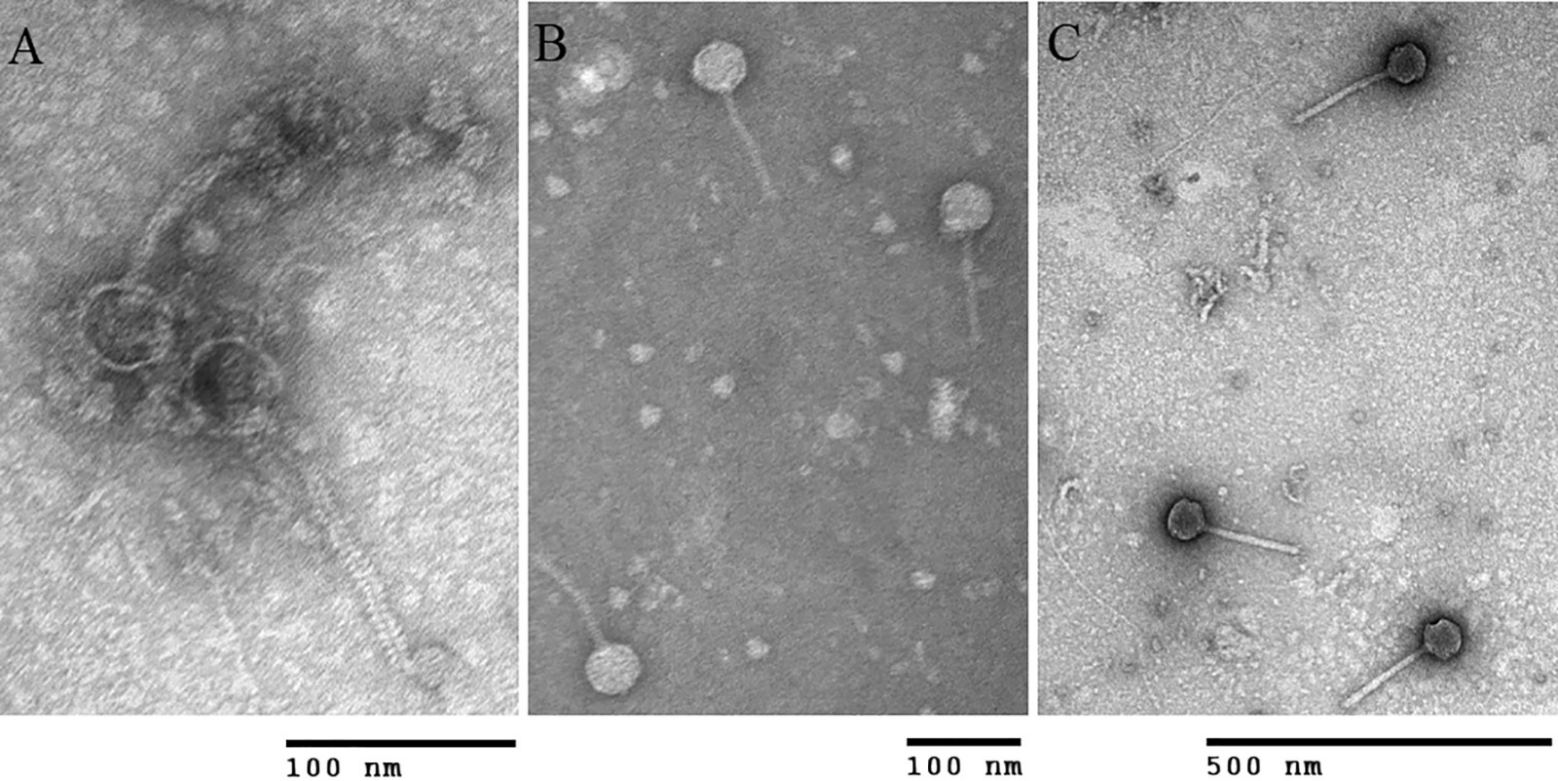
Transmission electron microscopy photos of Microbacterium foliorum bacteriophages Danno (A), Otwor (B), and Scumberland (C) showing *Siphoviridae* morphology with a long flexible tail. Bacteriophage samples were stained using 1% uranyl acetate on grids attached to Pelco tabs (Ted Peller, Inc., Redding, CA). The Hitachi H-7650 transmission electron microscope (Tokyo, Japan) was used with an accelerating voltage of 100 kV.

As part of bacteriophage diversity and geographical distribution studies, three new bacteriophages (Danno, Otwor, and Scumberland) were found in wet and warm (25°C) soil samples of Clarksville, TN (36.5343°N, 87.3525°W; 36.5322°N, 87.3523°W; and 36.541667°N, 87.367725°W, respectively), using the host bacterium Microbacterium foliorum NRRL B-24224. Bacteriophages were isolated using the enriched isolation technique as described in the Phage Discovery Guide (https://seaphagesphagediscoveryguide.helpdocsonline.com/home). We added soil samples to the peptone-yeast calcium medium for 2 h, passed the suspension through a 0.22-μm-pore filter, and incubated it with *M. foliorum* at 30°C on a shaker (250 rpm) for 2 days, and subsequently, we tested the sterile filtrate for bacteriophage plaque-forming units with the spot test (1). Bacteriophages were purified using the plaque assay and amplified to high-titer lysates by flooding “webbed” plates with phage buffer as described in the Phage Discovery Guide cited above. Bacteriophages were examined using transmission electron microscopy and found to be from the *Siphoviridae* family with a flexible tail and an icosahedral capsid (Fig. 1).

Bacteriophage DNAs were isolated and purified using a Promega Wizard DNA clean-up kit with spin washes (2). We prepared the genomic library using the New England Biolabs (NEB) Ultra II FS kit. The Pittsburgh Bacteriophage Institute sequenced the phage genomes using Illumina MiSeq technology with 150-base single-end reads, yielding approximate coverages of 2,631-fold for Danno (325,100 reads), 4,918-fold for Otwor (601,100 reads), and 73-fold for Scumberland (209,200 reads). The raw reads were assembled with Newbler v.2.9 and quality controlled and finished using Consed v.29. Genomic termini were determined as previously described (3). We manually annotated each genome using DNA Master v.5.23.6, GLIMMER v.3.02 (4), GeneMark v.2.5p (5), Phamerator v.393 (6), PhagesDB BLAST (https://phagesdb.org/blastp/) (7), NCBI BLAST (8), HHPred v.3.2 (9), and PECAAN (http://pecaan.kbrinsgd.org/). Default parameters were used for all software unless otherwise specified.

Bacteriophages Danno and Otwor are from cluster EE (10) with DNA sizes of 17,452 bp and 17,454 bp, respectively, and with GC content of 68.7%. Actinobacteriophage clusters are grouped together according to their nucleotide sequence similarity (11). The cluster EE contains the smallest actinobacteriophage genomes (3). Both bacteriophages have 25 predicted protein-coding genes (18 of which we identified the predicted function for). The gene content similarity (GCS), a measure of the proportion of genes two genomes share, for these two bacteriophages was 96% and was calculated using tools available on https://phagesdb.org/genecontent/. Both bacteriophages have predicted programmed translational frameshifts in genes 10 and 11 (tail assembly chaperone genes). Based on GCS, Danno and Otwor are closely related (96%) to bacteriophage BurtonThePup (found in Chevy Chase, MD). Bacteriophage Scumberland (53,276-bp genome size with 68.8% GC content) is from a different cluster, EC (10), and has 92 predicted protein-coding genes (27 of which we assigned predicted function for). Based on GCS, this bacteriophage is closely related to bacteriophage Selwyn23 (95.08%), found in Charlotte, NC. Scumberland has an 18-bp sequence (5′-TAGACTATAGGTGTAAGC) with unknown function, repeated nine times in its genome, similar to other EC cluster members (10). There are some variations in the repeat in our phage. We found a slightly different repeat (TAGgCTATAGGTGTAAGC) 27 bp upstream of a predicted translation initiation codon of gene 87.

### Data availability.

The accession numbers for Danno and Otwor are MT316462 and MT316459 (GenBank) and SRX10124534 and SRX10124535 (SRA), respectively. The GenBank and SRA accession numbers for Scumberland are MT818415 and SRX10124536, respectively.
